# MAPK/ERK and PI3K/AKT signaling pathways are activated in adolescent and adult acute lymphoblastic leukemia

**DOI:** 10.1002/cnr2.1912

**Published:** 2023-10-22

**Authors:** Gustavo Loureiro, Daniella M. Bahia, Maria Lucia M. Lee, Mair Pedro de Souza, Eliza Y. S. Kimura, Denise Carvalho Rezende, Marçal Cavalcante de Andrade Silva, Maria de Lourdes L. F. Chauffaille, Mihoko Yamamoto

**Affiliations:** ^1^ Division of Hematology Universidade Federal de São Paulo (EPM‐UNIFESP) São Paulo São Paulo Brazil; ^2^ Instituto de Oncologia Pediátrica Grupo de Apoio ao Adolescente e a Criança com Câncer (GRAACC) São Paulo São Paulo Brazil; ^3^ Division of Hematology Hospital Amaral Carvalho Jaú São Paulo Brazil

**Keywords:** ALL, ERK, MAPK, PI3K/Akt

## Abstract

**Background**: The mitogen‐activated protein kinase (MAPK)/ERK signaling cascade and the phosphoinosytol‐3 phosphate/Akt (PI3K/Akt) pathways are involved in proliferation and differentiation of hematopoietic cells. The frequency of PI3K/Akt and MAPK pathway activation in adult acute lymphoblastic leukemia (ALL) still need to be elucidated.

**Aims**: To assess the activity and prognostic implications of MAPK/ERK and PI3K/Akt pathways in adult (ALL).

**Methods:**

We examined 28 precursor‐B‐cell ALL and 6 T‐cell primary ALL samples. Flow cytometry was employed to analyze the expression levels of phosphorylated ERK and phosphorylated Akt.

**Results:**

Ten out of 15 (67%) ALL fresh samples (7 B‐cell, 3 T‐cell) showed constitutive p‐ERK expression. The p‐ERK mean fluorescent index ratio (MFI (*R*)) showed a tendency to be higher in ALL than in normal T lymphocytes (1.26 [0.74–3.10] vs. 1.08 [1.02–1.21], respectively [*p* = .069]) and was significantly lower than in leukemic cell lines (median MFI (*R*) 3.83 [3.71–5.97] [*p* < .001]). Expression of p‐Akt was found in 35% (12/34) (10 B‐cell, 2 T‐cell). The median MFI (*R*) expression for p‐Akt in primary blast cell was 1.13 (0.48–9.90) compared to 1.01 (1.00–1.20) in normal T lymphocytes (*p* = ns) and lower than in leukemic cell lines (median MFI (*R*) 2.10 [1.77–3.40] [*p* = .037]). Moreover, expression of p‐ERK was negatively associated with the expression of CD34 (1.22 [0.74–1.33] vs. 1.52 [1.15–3.10] for CD34(+) and CD34(−) group, respectively, *p* = .009).

**Conclusion:**

Our findings suggest that both MAPK/ERK and PI3K/Akt are constitutively activated in adult ALL, indicating a targeted therapy potential for ALL by using inhibitors of these pathways.

## 1 INTRODUCTION

Acute lymphoblastic leukemias (ALL) are clonal diseases caused by sequential acquisition of genetic alterations (mutations) in immature progenitors, resulting in differentiation disturbance leading to proliferation of immature lymphoid blast cells.^1^, ^2^ In contrast to the successful history of childhood leukemia treatment, with the survival rates around 90%, the treatment of adult ALL has only shown modest improvements over the last 2 decades, with the overall survival rates varying from 15% to 40%.^3^, ^4^

The normal hematopoietic process involves several signaling pathways that ensure normal cell survival. The phosphoinositide 3‐kinase (PI3K)/Akt and mitogen activated protein kinase (MAPK) signaling pathways stimulate mitosis, leading cells to proliferation and are also involved in inhibition of apoptosis, being both crucial in normal cell growth.^5^, ^6^, ^7^ They are also known to have a critical contribution on oncogenesis as they promote survival and proliferation of cancer cell including leukemia^8^ and solid tumors.^9^ One signal that is overactivated in a wide range of tumor types is the production of a phospholipid, phosphatidylinositol triphosphate, by phosphatidylinositol 3‐kinase (PI3K). This lipid and the activated protein kinase (Akt) trigger a cascade of responses, from cell growth and proliferation to survival and motility that drive tumor progression.^10^ It is also postulated that alterations in the bone marrow niche may contribute inducing activations of these pathways.^11^ In addition, PI3K/Akt and MAPK pathways have also been reported to be constitutively activated in leukemic blast cells from patients with acute myeloid leukemia (AML) and contribute to sustain proliferation, inhibit apoptosis, and elicit transformation.^12^, ^13^ Regarding to ALL, studies suggest that PI3K/Akt and MAPK pathways are also activated in both T‐ALL^14^, ^15^, ^16^ and B‐ALL.^14^, ^17^ Some studies in childhood ALL have shown an independent activation of both pathways in variable proportions.^18^, ^19^ The frequency of PI3K/Akt and MAPK pathway activation and its association with prognostic factors in adult ALL are still under investigation.^20^, ^21^

Importantly, components of these pathways have emerged as promising targets for the development of specific inhibitory compounds.^22^, ^23^ Drugs, such as perifosine,^24^ andrographolide,^25^ and PKI‐587,^26^ have shown anti‐leukemic effects through PI3K/Akt pathway blockade. In addition, drug sensitivity to the mitogen‐activated protein kinase (MEK) inhibitors has been shown to correlate with ERK activation status in AML.^13^ More recently, drugs such as proteasome inhibitors (bortezomib) have shown antileukemic effects in AML through inhibition of both pathways.^27^ In addition, it has been shown that MAPK pathway activation may contribute to resistance to PI3K inhibitors,^28^ evidencing the importance of crosstalk between those two pathways.

Because targeted therapeutics are envisioned also in ALL treatment, a rapid analysis of the activation status of the main signaling pathways in blast cells from ALL patients at diagnosis is required. Thus, this study was designed to evaluate the activation status of PI3K/Akt and MAPK/ERK pathways by flow cytometry in adolescent and adult patients with ALL and correlate with biological and clinical parameters, aiming to a better understanding of the biological behavior of these pathways in adult ALL.

## 2 PATIENTS AND METHODS

### 2.1 Patients

Thirty four patients, 25 adult (male = 15, female = 10) and nine adolescent (male = 7, female = 2) (median age 32.5 years, range 14–69 years) from São Paulo, Brazil, with newly diagnosed ALL were enrolled in this study. All patients gave their informed consent for the study which was provided according to the declaration of Helsinki. The study had approval of the ethical committee of the participant institutions. Diagnosis of ALL was based on blast cells morphology and immunophenotyping by multiparametric flow cytometry (MFC) in peripheral blood (PB) and/or bone marrow (BM) blast cells and ALL classification was based on the European Group for the Immunological Classification of Leukaemias (EGIL) criteria.^29^ There were 6 T‐ALL and 28 B‐ALL (3 B1 [pro‐B ALL], 10 B2 [common B‐ALL], 10 B3 [pre‐B ALL] and 5 B4 [mature or Burkitt type ALL]) cases (Table S1). The treatment protocols used were the Brazilian Treatment Group for Leukemias in Childhood (GBTLI) for adolescents (14–16 years)^30^ and the German multicenter ALL (GMALL 5/93) protocol for adults (>16 years),^31^ consisting both of a 7‐days pre‐phase (prednisone 20 mg/m^2^ and Vincristine 2 mg) followed by standard induction chemotherapy.

### 2.2 Materials and methods

#### 2.2.1 Primary samples

BM samples were collected in ethylenediaminetetraacetic acid (EDTA) at diagnosis and at the end of induction therapy for measurable/minimal residual disease (MRD) detection. PB samples were also used in BM dry‐tap (failure to obtain bone marrow on attempted marrow aspiration) and/or in very high white blood cell (WBC) count cases. Blast cells were enriched by Ficoll–Hypaque® (#GE17‐1440‐02, 1077 g/mL; Sigma, Milan, Italy) gradient centrifugation and used for the phosphorylation assays. Previously dimethylsulphoxide (DMSO) cryopreserved diagnostic leukemic cells were used for 19 cases. Samples with >80% of viable cells after thawing process were used.

#### 2.2.2 Evaluation of p‐ERK and p‐Akt expression by MFC

Expression of p‐ERK and p‐Akt was evaluated by MFC using a previously described procedure^24^ with modifications. Briefly, cells either untreated or treated with mitogen‐activated protein kinase kinase (MEKK) inhibitor, U0126 10 mM (#9903, Cell Signaling, Beverly, MA, USA) working solution in methanol (#A411‐20, Fisher Scientific, Fair Lawn, NJ, USA), and phorbol myristate acetate (PMA) 40 μM working solution from 40 mM stock in ethanol (#493511, Sigma‐Aldrich, St. Louis, MO, USA), a potent inductor of ERK phosphorylation, were fixed using 2% formaldehyde (#18814‐10, Polysciences Inc., PA, USA) at 37°C for 10 min. Samples were then resuspended in 90% ice‐cold methanol (#A411‐20, Fisher Scientific, Fair Lawn, NJ, USA) and kept on ice for 30 min, washed once with RPMI medium and then labeled with alexafluor488‐conjugated p‐ERK (#9106) and rabbit p‐Akt Ser473 (#4058) monoclonal antibodies (Cell Signaling Technology, Beverly, MA, USA) for 15 min at room temperature, according to the manufacturer's instruction. Data acquisition and analysis were performed using a FACScalibur flow cytometer (Becton Dickinson Bioscience, San Jose, CA, USA) and CellQuest software (BDB). Phospho‐ERK was considered activated by PMA or inhibited by U0126 when the expression increased or reduced in ≥25%, respectively.^32^

As controls, MAPK and PI3K/Akt pathways were also evaluated in PB T‐cells from 5 and 3 healthy donors, respectively, in T‐cell leukemia cell lines (Jurkat and CEM) and in a myeloid cell line (HL60). Normal steady‐state CD3+ lymphocytes are known to be negative or express very low levels of p‐ERK and p‐Akt,^33^, ^34^ while Jurkat, CEM, and HL‐60 cell lines express p‐ERK and p‐Akt at variable levels.^35^

#### 2.2.3 Cytogenetic and molecular analysis

Cytogenetic analysis was performed following standard G‐band technique.^36^ Cases with hyperdiploidy (50–60 chromosomes) were considered favorable prognosis, normal karyotype and isolated 9p/*p15‐p16* were classified as a “standard‐risk” group, whereas patients with *t*(9;22), *t*(4;11), and *t*(1;19) as an “adverse risk” category. Patients with del(6q) and miscellaneous abnormalities, were included in the “intermediate risk” group.^37^ In all precursor B‐ALL cases (except B4), *BCR‐ABL1* and *MLL(KTM2A)‐AF4* fusion transcripts were investigated by RT‐PCR using RNA samples extracted from BM or PB mononuclear cells at diagnosis. To identify the common fusion genes in ALL—namely, *MLL‐AF4*, *BCR‐ABL1* p190 and p210 isoforms—we used a standardized RT‐PCR protocol defined by the Biomed‐1 Concerted Action.^38^

#### 2.2.4 P‐glycoprotein (P‐gp) expression and function

Multi‐drug resistance (MDR) was evaluated through expression and function of the glycoprotein P (P‐gp). P‐gp expression was evaluated using MDR1 monoclonal antibody (#557003, BD, San Jose, CA) according to previously described procedure^39^ and was considered positive when 20% or more blasts expressed the antigen.^40^ P‐gp function analysis was investigated using the rhodamine‐123 (Rhd‐123) efflux test, according to a previously described procedure.^41^

#### 2.2.5 Detection of the MRD by flow cytometry

Using the aberrant phenotypes detected at diagnosis, the MRD investigation was performed in bone marrow samples at the end of induction I (D28). The technique has been previously described.^42^ The final calculation of residual cells was performed in total nucleated cells of the sample and the MDR was considered positive when >0.01%.

#### 2.2.6 Statistical analysis

The p‐ERK and p‐Akt expression levels were analyzed using the ratio (*R*) between the mean fluorescence intensity (MFI) of primary sample and the control isotype by flow cytometry technique. The Mann–Whitney test was used to evaluate the differences between groups. Results are expressed as median and range. The Pearson correlation test was used to determine whether cryopreserved and fresh sample groups were comparable. Values of *p* < .05 were considered statistically significant.

## 3 RESULTS

### 3.1 Evaluation of p‐ERK and p‐Akt levels in fresh and cryopreserved ALL samples

To evaluate the effect of DMSO cryopreservation in liquid nitrogen, we compared the expression pattern of phosphorylated proteins in fresh and cryopreserved samples of the same patient in 5 consecutive ALL cases.

Cells either fresh or cryopreserved ones were stained for p‐ERK and p‐Akt and phospho‐protein expression was evaluated side by side. Phospho‐ERK expression was different in cryopreserved and fresh samples (fresh vs. cryopreserved) (*r* = −.53, *p* = .35, Pearson test) but regarding p‐Akt there was a tendency to correlation (*r* = .85, *p* = .06, Pearson test) between groups (Supplemental Table S2). Response of MAPK/ERK pathway to PMA stimulus showed higher frequency of p‐ERK increase in fresh samples, in contrast to (60% vs. 16%) to cryopreserved ones (data not shown). Thus, the phospho‐proteins expression was evaluated only in fresh samples for the MAPK/ERK pathway (*n* = 15) and both groups (fresh and cryopreserved, *n* = 34) together for the PI3K/Akt pathway.

### 3.2 Evaluation of p‐ERK and p‐Akt levels in normal PB CD3+ cells and its response to PMA stimulus

**FIGURE 1:**
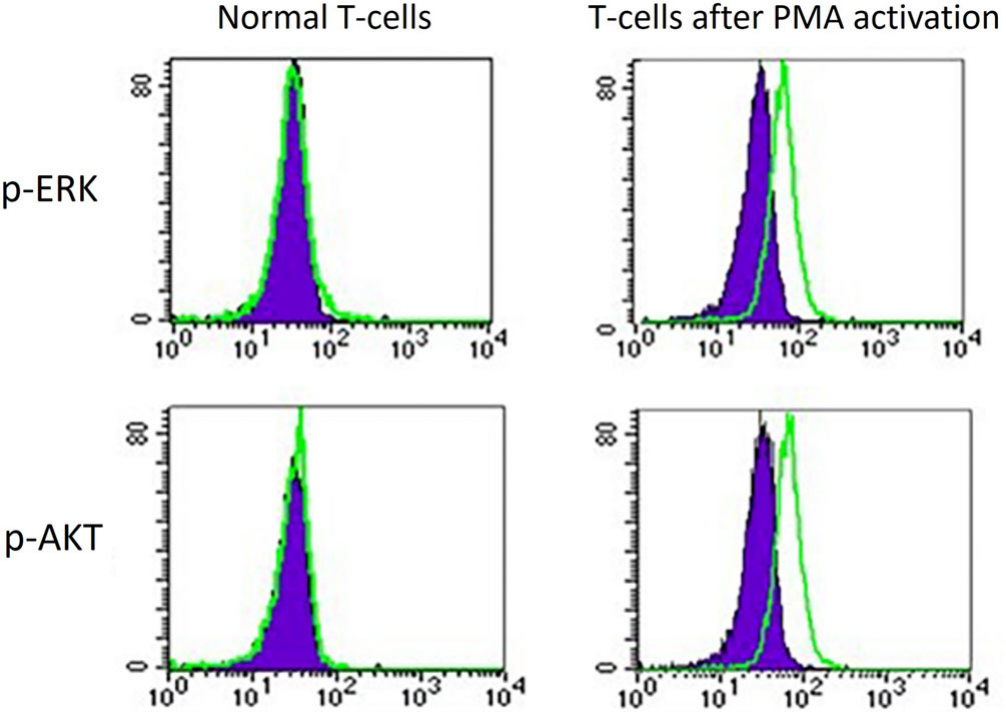
Phospho‐flow analysis of p‐ERK and p‐AKT expression, in histogram plots, on T lymphocytes, before and after PMA treatment (green line). Expression of the control isotype (purple line).

PMA treatment induced ERK and Akt phosphorylation in resting T‐lymphocytes (Figure 1). The median value of p‐ERK MFI (*R*) for steady‐state PB CD3+ lymphocytes was 1.08 (1.02–1.21) (*n* = 5), which increased when stimulated by PMA (1.98 [1.15–3.36]). Regarding p‐Akt the median MFI (*R*) was 1.01 (1.0–1.2) (*n* = 3) for steady‐state PB T‐lymphocytes and there was also increase after PMA stimulus (median 1.5 [1.21–2.16]).

### 3.3 Evaluation of p‐ERK and p‐Akt levels in normal PB CD3+ cells, cell lines and primary blast cells

#### 3.3.1 Normal PB CD3+ cells and cell lines

**FIGURE 2:**
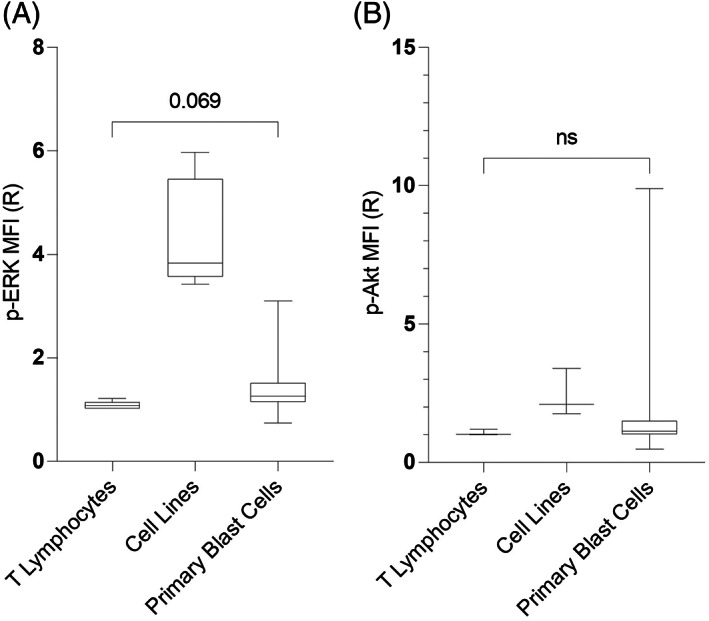
Mean florescence intensity (MFI) ratio (*R*) of (A) p‐ERK expressions (median and range) on resting T‐lymphocytes (1.1 [1.0–1.2]), cell lines (3.8 [3.4–6.0]), and primary blast cells (1.3 [0.74–3.1]), (B) p‐AKT expression on T‐lymphocytes (1.1 [1.0–1.2]), cell lines (2.1 [1.8–3.4]), and leukemic blast cells (1.3 [0.48–9.9]), respectively.

The leukemic cell lines expressed the highest expression of p‐ERK (3.83 [3.43–5.97]) which was higher than in steady state of control T‐lymphocytes (*p* = .008, Mann–Whitney test) (Figure 2A).

Phospho‐akt median value expression in cell lines was apparently higher than in PB T‐cells although not significant, possibly due to small number of studied cases (*p* = .1, Mann–Whitney test) (Figure 2B).

A cut‐off point was set based on the maximum expression on controls (PB T‐lymphocytes), being the values of 1.21 for p‐ERK and 1.20 for p‐Akt used to discriminate positive from negative primary ALL samples.

#### 3.3.2 Primary ALL samples

The median expression of p‐ERK MFI (*R*) in 15 fresh ALL samples was found to be 1.26 (0.74–3.10), which tended to be higher when compared to steady‐state peripheral blood (PB) CD3+ lymphocytes (*p* = .069, Mann–Whitney test). By using 1.21 as the cut‐off value, constitutive ERK phosphorylation was observed in 7 out of 11 (64%) B‐ALL and 3 out of 4 (75%) T‐ALL fresh sample cases (Table 1).

**TABLE 1:** Constitutive ERK and Akt phosphorylation in primary B‐ALL and T‐ALL blast cells.

	p‐ERK^a^		p‐Akt^b^	
	Positive	Negative	Positive	Negative
B‐ALL	7/11	4/11	10/28	18/28
T‐ALL	3/4	1/4	2/6	4/6

*Note*: A total of 15 fresh samples were analyzed for p‐ERK and 34 cryopreserved and fresh for p‐AKT status.

^a^ Cut‐off *R* > 1.21.

^b^ Cut‐off *R* > 1.20.

The median MFI (*R*) of p‐Akt expression in 34 samples was 1.13 (0.48–9.90), being similar to T‐lymphocytes (1.01 [1.00–1.20]) and lower than in cell lines (2.1 [1.7–3.4]) (*p* = .037, Mann–Whitney test). Considering *R* = 1.20 as a cut‐off for p‐Akt expression, constitutive Akt phosphorylation was observed in 10 out 28 (36%) B‐ALL and 2 out 6 (33%) T‐ALL (Table 1).

Evaluation of activation (PMA) and inhibition (U0126) of p‐ERK showed a positive response to PMA in 60% of leukemic cells. Inhibition (≤25%) was observed in 3/15 (20%) samples. Nevertheless, when we considered any reduction or when *R* became <1.21 (cutoff value) 60% (9/15) of the cases had inhibited p‐ERK expression.

In 4 patients both p‐ERK (fresh sample group) and p‐AKT were simultaneously activated. In this particular subgroup, median p‐ERK expression was 1.29 (1.24–1.52) and median p‐AKT expression was 1.475 (1.27–1.96). All four cases were B‐ALL (2 B3, 1 B3, and 1 B4).

### 3.4 Association between p‐ERK and p‐Akt levels and ALL immunological subtypes, clinical and biological parameters

#### 3.4.1 ALL EGIL classification

No difference of p‐ERK or p‐AKT activation was observed between overall fresh B‐cell ALL (1.26 [0.74–3.10]) and T‐cell ALL (1.54 [1.01–5.94]) (*p* = .31, Mann–Whitney test). The frequency of activation of the p‐ERK pathway was 75% (9/12) for B‐cell ALL and in one studied case of T‐cell ALL (MFI (*R*) = 1.52). Both p‐ERK and p‐AKT pathways were activated in most of Burkitt leukemia (subtype B4) (p‐AKT = 4/5, 80% and 1/1 p‐ERK) (Supplemental Table S3).

#### 3.4.2 CD34 antigen on leukemic cell

p‐ERK expression correlated negatively to the CD34 expression [p‐ERK 1.22 (0.74–1.33) vs. p‐ERK 1.52 (1.15–3.10) in CD34 positive and negative cells, respectively (*p* = .009), Mann–Whitney test. There was no relationship between p‐Akt expression and CD34 or CD10 positivity.

#### 3.4.3 Other clinical‐biological parameters

Considering multi‐drug resistance, 30 cases were evaluated. Of these, 12 (40%) had rhodamine efflux that was inhibited by verapamil (*R* value ≥1.10), demonstrating GPP‐mediated MDR. However, there was no difference in the p‐ERK and p‐Akt expression between the MDR positive and MDR negative groups. Those MDR positive cases presented lower response to the stimulus (PMA) and inhibition (U0126) compared with MDR negative cases (data not shown). Regarding minimal residual disease detection, it was possible to look for MRD in 13 patients who achieved complete morphologic remission after induction therapy, and that had aberrant phenotypes that could be detected. In these cases, MRD was positive (>0.01%) in 5 patients (38.4%) (4 B‐ALL and 1 T‐ALL), but also no difference was observed regarding p‐ERK and p‐Akt expression between those groups.

Considering the particular subgroup of four B‐ALL cases where both p‐ERK and p‐AKT pathways were activated, it was also not possible to associate with phenotypic, clinical or biological parameters analyzed.

Moreover, no association was found between p‐ERK or p‐Akt expression and the patients age, gender, WBC count and cytogenetic abnormalities (data not shown), possibly due to the small number of cases analyzed.

## 4 DISCUSSION

The MAPK/ERK and PI3K/Akt cell signaling pathways play an important role in directing the development, proliferation, and cell survival,^43^ through the activation of proteins that mediate the proliferation (p‐ERK and p‐Akt) and cellular apoptosis (p‐Akt).^44^, ^45^ Recent studies show activation of these pathways in various cancers.^46^, ^47^, ^48^ The constitutional activation of MAPK/ERK pathway is often found in solid tumors, for example, rhabdomyosarcoma, colon and lung cancers and melanoma,^20^, ^49^, ^50^ as well as in hematologic neoplastic diseases, particularly in AML.^17^, ^32^, ^51^ Huang et al.^51^ have found that aberrant MAPK/ERK expression in pluripotent cells is involved in leukemic transformation in mice. Using liquid chromatography coupled tandem mass spectrometry it was possible to identify phosphorylated serines on AML cells that were abundantly phosphorylated in leukemic cells but not in healthy hematopoietic stem and progenitor cells (HSPCs). In ALL, the role of the p‐ERK signaling pathway have been studied.^14^, ^52^, ^53^ Towatari et al.^53^ were the first to study the MAPK/ERK pathway in patients with ALL and found that it was activated in 3 out of 9 B‐ALL cases, but none of four T‐ALL cases. Later studies by Meng et al.^52^ in 24 patients and Gregorj et al.^14^ in 131 cases showed activation of this pathway in about one third of cases of B‐ALL. In our current study, we found constitutive activation of MAPK/ERK pathway in two thirds (66%) of cases, B and T cell ALL considered. This suggests the involvement of this pathway in the leukemogenesis process, as shown in AML and other neoplasms. PI3K/Akt pathway, however, was activated less frequently, however we used fresh and frozen samples and could lead to misinterpretation expression.

It is worth emphasizing the importance of comparative studies conducted between fresh and cryopreserved samples from the same patient, since we detected different results of p‐ERK expression in these two conditions, with a potential interference of freezing and thawing process, although some studies use fresh and frozen/thawed cells indistinctly.^54^, ^55^ This hypothesis was corroborated by the findings during the test activation with PMA stimulus, since there was less frequent activation of this protein among cryopreserved samples as compared to fresh ones, suggesting this signaling pathway may be sensitive to the effects of cryopreservation procedure. It may have altered the expression of intracellular proteins, also making them less sensitive to drug treatment in vitro. For p‐Akt, the results of fresh and frozen samples were analyzed together, since there was a trend toward correlation between them.

Although a small sample of cases here studied, we found a high positivity for p‐ERK in B‐ALL. Our data are comparable with the frequency found for AML and higher than that found in ALL by Gregorj et al.^14^ in fresh samples. It is noteworthy that although these authors have also used the technique of multiparametric flow cytometry, they used different phospho‐marking and analysis approach. We used direct labeling method, which reduces test time and number of cell washing compared to the indirect labeling.^14^ In addition, data analysis was performed using the Kolmogorov–Smirnov (KS) test which compares the expression of the fluorochrome p‐ERK with the isotype control, considering positive when the *D* value was ≥0.10. In our results, we used the relationship between the mean fluorescence intensity values of patient samples and negative controls. Therefore, the labeling method and the statistical approach to referring positive results might explain these differences, in addition to the sample size. Other studies have used fresh and previously frozen sample possibly because of small differences observed in pathway activation status. However, in our series, it was observed that MAPK/ERK activation may be influenced by the freezing/thawing process.

Albeit in our series it was not possible to verify a conclusive statistical higher expression of p‐Akt in cell lines when compared to T‐lymphocyte controls possibly because of the small sample number, our results show that the PI3K/Akt pathway is also active in ALL, although at a lower frequency (35%), in both T‐ (30%) and in B‐ALL (36%). This pathway has been most studied in AML and T‐ALL, in which p‐Akt is constitutively expressed in most cases.^56^, ^57^, ^58^, ^59^, ^60^ This pathway has also been studied in relation to the PTEN gene changes, showing that mutations occur in approximately a half of cases of T‐ALL in children,^15^ although less (12%) in adult population.^61^

Comparing the state of activation of MAPK pathway with other clinical and laboratory parameters, Gregorj et al.^14^ found an association between high WBC count (>30 × 10^9^/L) and activation of the MAPK/ERK. We could not find this relationship, possibly due to the small number size of our series. Likewise, it was not possible to establish a relationship with the subtypes of ALL, T or B, nor with major cytogenetic abnormalities associated with poor prognosis in ALLs. Interesting, however, was the observation of PI3K/Akt pathway activation in four out of five B4‐ALL (Burkitt leukemia) cases. This pathway, apparently, has more relevance in the pathophysiology of B4 ALL in which the clinical, cytogenetic, and molecular characteristics are distinct from other B‐ALLs. Thus, we can suggest that PI3K/Akt pathway has a great relevance in the pathophysiology in B4 and may have a potential role for Burkitt therapy. Further studies with larger number B4 cases are required for confirmation.

Another interesting finding was the inverse correlation between CD34 antigen and the p‐ERK expressions. This negative association confirms the data reported by Gregorj et al.^14^ Normal bone marrow hematopoietic CD34+ stem cells usually do not express p‐ERK. However, when considering acute leukemias and cancers that originate from transformation of more immature progenitor cells largely preserving the properties of the “original” cell phenotype, it would be expected that this signaling pathway would be also active in CD34‐positive ALL.

This finding, along with other observations, leads to uncertainty as to the nature of the leukemic stem cell, one of the most important focus of attention today. More recent studies involving single‐cell proteomics and genomics, demonstrate the existence of different populations of leukemic clones.^62^, ^63^, ^64^ Thus, it would be interesting to study the activation of this pathway in leukemic stem cells using, for example, xenotransplantation models. This could contribute to the elucidation of the role of this pathway in leukemogenesis process. To assess the implications of the activation of these pathways in the prognosis of ALL, we studied their relationship with the level of MRD detection during therapy, considered the most important prognostic factor in this disease, overcoming several other prognostic variables.^65^ With respect to the MAPK/ERK pathway, we highlight the fact that MRD+ patients (4/4) presented low expression of p‐ERK. The MAPK/ERK pathway is responsible for recruiting proliferative signal, leading to increased cell proliferation. Thus, it is suggested that cases with positive expression of p‐ERK would be more susceptible to chemotherapeutic action and therefore had lower MRD. Concerning the response to chemotherapy Gregorj et al.^14^ showed a greater likelihood of remission at 4 weeks in patients who did not express p‐ERK compared to the way they were constitutively active. Our results agree with their findings although they did not evaluate the ERK pathway in the context of MRD.

As demonstrated in our study and others, the high frequency of activation of MAPK/ERK in ALL and PI3K/Akt, specifically in B4 subtype, shows that deregulation of these pathways are key events in the pathogenesis of leukemia, making these proteins candidates for regulatory targeted therapies. The action of drugs in this cell signaling pathway has been shown a therapeutic potential in lymphoproliferative disorders such as acute lymphoid leukemia.^66^, ^67^ In this sense, testing with inhibitors of these pathways may be carried out, possibly showing inhibition of proliferation of leukemic cells, leading them to apoptosis.

Our findings are in agreement with other authors, reinforcing the relevance of MAPK and AKT in ALL pathogenesis and potential therapeutic targets. We carefully conducted pathway evaluation status, controlling for freezing and thawing of cells that interfere in these evaluation, as shown by us. Therefore we should be aware of these limitations when interpreting those data. The small sample size may jeopardize statistical analysis, as was not possible to correlate many clinical‐biological features with altered constitutional antiapoptotic pathways. We have also analyzed only two of many antiapoptotic proteins and pathways that maybe also altered in the context of ALL. For a larger sample size the percentage of activation of those pathways almost certainly would vary. In addition, we have not analyzed the molecular counterparts of those pathways, which may contribute to a better understanding of causality.

In conclusion, our results demonstrate the relevance of these pathways in the process of leukemogenesis in ALL. Thus, MAPK/ERK and PI3K/Akt pathways show a potential role for the development of specific targeted therapy, contributing to the management of adult ALL patients.

## AUTHOR CONTRIBUTIONS

**Gustavo Loureiro**: Conceptualization (equal); formal analysis (lead); investigation (equal); writing—original draft (equal); writing—review & editing (equal). **Daniella M. Bahia**: Supervision (supporting); writing—original draft (equal); writing—review & editing (equal). **Maria Lucia M. Lee**: Resources (equal). **Mair Pedro de Souza**: Resources (equal). **Eliza Y. S. Kimura**: Investigation (equal). **Denise Carvalho Rezende**: Investigation (equal). **Marçal Cavalcante de Andrade Silva**: Investigation (equal). **Maria de Lourdes L. F. Chauffaille**: Resources (equal); writing—review & editing (equal). **Mihoko Yamamoto**: Conceptualization (equal); supervision (lead); funding acquisition (lead) writing—original draft (equal); writing—review & editing (equal).

## FUNDING INFORMATION

This paper was supported by a grant from the Brazilian agency Fundação de Amparo à Pesquisa do Estado de São Paulo (FAPESP—São Paulo Research Foundation, Proc. Nr. 2009/51002‐8).

## CONFLICT OF INTEREST STATEMENT

The authors have stated explicitly that there are no conflicts of interest in connection with this article.

## Supporting information


**Supplemental Table S1** Distribution of ALL patients according to the EGIL classification, age, and WBC.
**Supplemental Table S2** Evaluation of p‐ERK and p‐AKT levels in fresh and cryopreserved samples.
**Supplemental Table S3** Frequency of p‐ERK and p‐AKT expression according to immunological subtypes.

## Data Availability

Data available on request from the authors.
