# Supplementary material for: MAPK/ERK and PI3K/AKT signaling pathways are activated in adolescent and adult acute lymphoblastic leukemia
Source: Cancer Rep (Hoboken). 2023 Oct 22;6(12):e1912. doi: 10.1002/cnr2.1912 (PMC10728523; doi:10.1002/cnr2.1912)
Supplement: Supplementary file 1 — Supplemental Table S1 Distribution of ALL patients according to the EGIL classification, age, and WBC. Supplemental Table S2 Evaluation of p‐ERK and p‐AKT levels in fresh and cryopreserved samples. Supplemental Table S3 Frequency of p‐ERK and p‐AKT expression according to immunological subtypes. [file CNR2-6-e1912-s001.docx]

SUPPLEMENTAL MATERIALS

**Supplemental table S-1.** Distribution of ALL patients according to the EGIL classification, age, and WBC.

| **EGIL Classification** | **n (%)** | **Age years**  **Median (range)** | **WBC (x10^9^/L)**  **Median (range)** |
| --- | --- | --- | --- |
| B1 | 3 (9) | 21 (16-28) | 84.7 (12.3-280.0) |
| B2 | 10 (29) | 32.5 (14-60) | 60.0 (2.3-373.0) |
| B3 | 10 (29) | 54 (14-69) | 18.0 (1.5-175.0) |
| B4 | 5 (15) | 33 (19-62) | 14.8 (12.1-21.9) |
| T | 6 (18) | 23 (17-56) | 17.0 (6.0-99.0) |
| TOTAL | 34 | 32 (14-69) | 19.3 (1.5-373.0) |

**Supplemental table S-2.** Comparison of the p-ERK and p-AKT expression levels (MFI-R) in fresh and cryopreserved samples.

| **Samples** | **Fresh** | **Cryopreserved**  **in DMSO** | | | **p*** |  |
| --- | --- | --- | --- | --- | --- | --- |
| **p-ERK**  1 | 1.26 | | 2.07 |  | | |
| 2 | 0.86 | | 3.33 | r=-0.53, | | |
| 3 | 1.52 | | 3.51 | p=0.35 | | |
| 4 | 1.48 | | 1.41 |  | | |
| 5 | 1.60 | | 1.10 |  | | |
| Median  (range) | 1.48  (0.86-1.60) | | 2.07  (1.10-3.51) |  | | |
| **p-AKT**  1 | 1.66 | | 3.88 |  | | |
| 2 | 6.07 | | 5.26 | r=0.85 | | |
| 3 | 1.96 | | 2.92 | p=0.06 | | |
| 4 | 0.91 | | 1.27 |  | | |
| 5 | 0.89 | | 0.99 |  | | |
| Median  (range) | 1.66  (0.89-6.07) | | 2.92  (0.99-5.26) |  | | |

* Pearson correlation test

**Supplemental table S-3**. Frequency of constitutively activated p-ERK and p-AKT expression according to immunological subtypes.

|  |  | Elevated p-ERK |  | Elevated p-Akt |  |
| --- | --- | --- | --- | --- | --- |
| Phenotype | EGIL | n | n (%) | n | n (%) |
| B-ALL | B1 | 0 | 0 | 3 | 0 |
|  | B2 | 5 | 5 (100) | 10 | 4 (40) |
|  | B3 | 5 | 1 (20) | 10 | 2 (20) |
|  | B4 | 1 | 1 (-) | 5 | 4 (80) |
| T-ALL | n | 4 | 3 (75) | 6 | 2 (33) |
| Total |  | 15 | 10 (66.6) | 34 | 12 (35.3) |

Comparison between B-ALL and T-ALL immunological subgroups was ns.
